# Comprehensive Transcriptome Analysis of Hair Follicle Morphogenesis Reveals That lncRNA-H19 Promotes Dermal Papilla Cell Proliferation through the Chi-miR-214-3p/β-Catenin Axis in Cashmere Goats

**DOI:** 10.3390/ijms231710006

**Published:** 2022-09-02

**Authors:** Yuelang Zhang, Fang Li, Yujie Shi, Tongtong Zhang, Xin Wang

**Affiliations:** Key Laboratory of Animal Genetics, Breeding and Reproduction of Shaanxi Province, College of Animal Science and Technology, Northwest A&F University, Yangling, Xianyang 712100, China

**Keywords:** cashmere goat, hair follicle morphogenesis, H19, chi-miR-214-3p, dermal papilla cells

## Abstract

Cashmere is initiated and develops in the fetal stages and the number and density of secondary hair follicles (SHFs) determine cashmere production and quality. Growing evidence indicates that both microRNA (miRNA) and long non-coding RNA (lncRNA) play an indispensable role in hair follicle (HF) growth and development. However, little is known about miRNAs, lncRNAs, and their functions as well as their interactions during cashmere initiation and development. Here, based on lncRNA and miRNA high-throughput sequencing and bioinformatics analysis, we identified 10,485 lncRNAs, 40,639 mRNAs, and 605 miRNAs in cashmere goat skin during HF induction, organogenesis, and cytodifferentiation stages. Among them, 521 lncRNAs, 5976 genes, and 204 miRNAs were differentially expressed (DE). KEGG analysis of DE genes indicated that ECM–receptor interaction and biosynthesis of amino acids were crucial for HF development. Notch, TGF-beta, and Wnt signaling pathways were also identified, which are conventional pathways associated with HF growth and development. Then, the ceRNA regulatory network was constructed, and the impact of lncRNA H19 was investigated in dermal papilla (DP) cells. The MTT, CCK-8, and EdU assays showed that the viability and proliferation of DP cells were promoted by H19, and mechanistic studies suggested that H19 performed its function through the chi-miR-214-3p/β-catenin axis. The present study created a resource for lncRNA, miRNA, and mRNA studies in cashmere morphogenesis. It could contribute to a better understanding of the molecular mechanism of ncRNAs involved in the regulation of HF growth and development.

## 1. Introduction

Cashmere is a precious textile produced by the secondary hair follicles (SHFs) of cashmere goat skin with the characteristics of good elasticity, strong moisture absorption, and effective heat preservation [1]. The Shanbei white cashmere goat is the predominant breed for the production of both cashmere and meat in the northern parts of Shaanxi province in China. These goats produce high yields of cashmere with a slender fiber, and they are highly adaptable. Cashmere goat hair follicles (HFs) include primary hair follicles (PHFs) and SHFs. The quantity and density of SHFs affect the yield and diameter of cashmere fibers determining the value of cashmere. Therefore, it is of economic interest to uncover the process and molecular mechanism of morphogenesis of cashmere in order to obtain higher cashmere yields per goat.

HFs originate from the interaction between the epidermis and dermis during the embryonic stage, and according to morphological structure and development time, the growth of goat hair follicles can be divided into three main stages: induction (~embryonic day 55–65), organogenesis (~embryonic day 85–95), and cytodifferentiation (~embryonic day 115–125) [2,3,4,5]. The induction phase includes the formation of placodes (PCs) and dermal condensates (DCs), with signals from the epithelium inducing the formation of the latter [6]. Organogenesis is the key phase for HF structure formation. HFs grow into more complete hair germs, or hair pegs, and are gradually differentiated into various types of cells: DC cells differentiate into dermal papilla (DP), while hair follicle stem cells (HFSC) gradually differentiate into outer root sheath cells (ORS) and matrix cells (Mx). Cytodifferentiation is the last stage of hair follicle development, and the formation of the inner root sheath (IRS), hair shaft, sebaceous gland, and arrector pili muscle denotes the maturation of HFs. Evidence from humans and mice indicates that some molecular signals are involved in the process of HF morphogenesis, including noggin, Wnt, sonic hedgehog (SHH), bone morphogenetic proteins (BMP), fibroblast growth factor (FGF), anhidrotic ectodermal dysplasia (Eda), and transcription factors [7,8,9,10,11,12].

It has reported that the miRNA-processing enzymes Dicer and Drosha are essential for the morphogenesis and maintenance of HFs [13,14]. Many miRNAs have been identified and characterized in the HF biology of mice. MiR-214 regulates skin morphogenesis and HF cycling by targeting β-catenin, and its overexpression inhibits the proliferation of keratinocytes, resulting in the formation of fewer HFs, smaller hair bulbs, and less hair production [15]. MiR-22 promotes the anagen-to-catagen transition of HFs and directly represses numerous upstream transcription factors of phenotypic keratin genes, including *Dlx3*, *Foxn1*, and *Hoxc13* [16]. LncRNAs are a class of non-coding RNAs with a length greater than 200 nt that are essential for the regulation of hair growth and the HF cycle [17]. Lin et al. found that the lncRNAs RP11-766N7.3, H19, and HOTAIR are abnormally expressed in DP cells at different growth stages, and they may affect the Wnt signaling pathway during the transition of HFs from telogen to anagen [18]. LncRNA-000133, a lncRNA that is more highly expressed at anagen rather than at telogen, led to a significant increase in relative expressions of the inductive property genes of *ET-1*, *SCF*, *ALP*, and *LEF1* in dermal papilla cells [19]. Overall, lncRNAs, as well as miRNAs, play an important role in the regulation of HF growth and development.

Cashmere is produced by SHFs, and its development during HF morphogenesis is essential for cashmere yield and quality. Although many miRNAs and lncRNAs have been identified in the cashmere goat hair cycle [20,21], little is known about their function and regulation mechanism in HF morphogenesis development. In the current study, the coding genes, lncRNAs, and miRNA profile of cashmere goat skin in cashmere induction, organogenesis, and cytodifferentiation were detected using RNA transcription sequencing. Subsequently, the function and regulatory mechanisms of the identified lncRNA H19 and miR-214 in DP cells were further explored. This study could improve the understanding of HF growth and development in cashmere goats.

## 2. Results

### 2.1. Identification of lncRNAs and miRNAs in Goat Skin

For lncRNA sequencing, nine transcriptomes were examined, covering the three different developmental stages of cashmere using the stranded sequencing method. In total, 874,836,744 raw reads were produced on the Illumina HiSeq 4000 platform (Illumina, San Diego, CA, USA). Then, 864,389,162 clean reads were obtained for downstream analysis. At the same time, Q20, Q30, and GC content of clean data were calculated (Table S1). Subsequently, we mapped the clean reads to the goat reference genome v2.0 using the HISAT2–Stringtie pipeline. After coding potential analysis using the software CNCI, CPC, and Pfam-scan, 2227 known lncRNAs and 8258 novel lncRNAs were identified (Supplementary File S2). At the same time, 40,364 known mRNAs and 275 novel mRNAs were identified (Supplementary File S3). For miRNA sequencing, a total of 177,780,733 clean reads were obtained (Table S2). Of these, 422 known miRNAs and 183 novel miRNAs were identified (Supplementary Files S4 and S5). The DE ncRNAs and genes from the induction, organogenesis, and cytodifferentiation stages were calculated by FPKM or TPM. As a result, 521 DE lncRNAs, 5976 DE genes, and 204 DE miRNAs were identified. A summary of the identified RNAs is provided in Table 1.

### 2.2. Differentially Expressed Analysis and lncRNA Target Prediction

**Figure 1:**
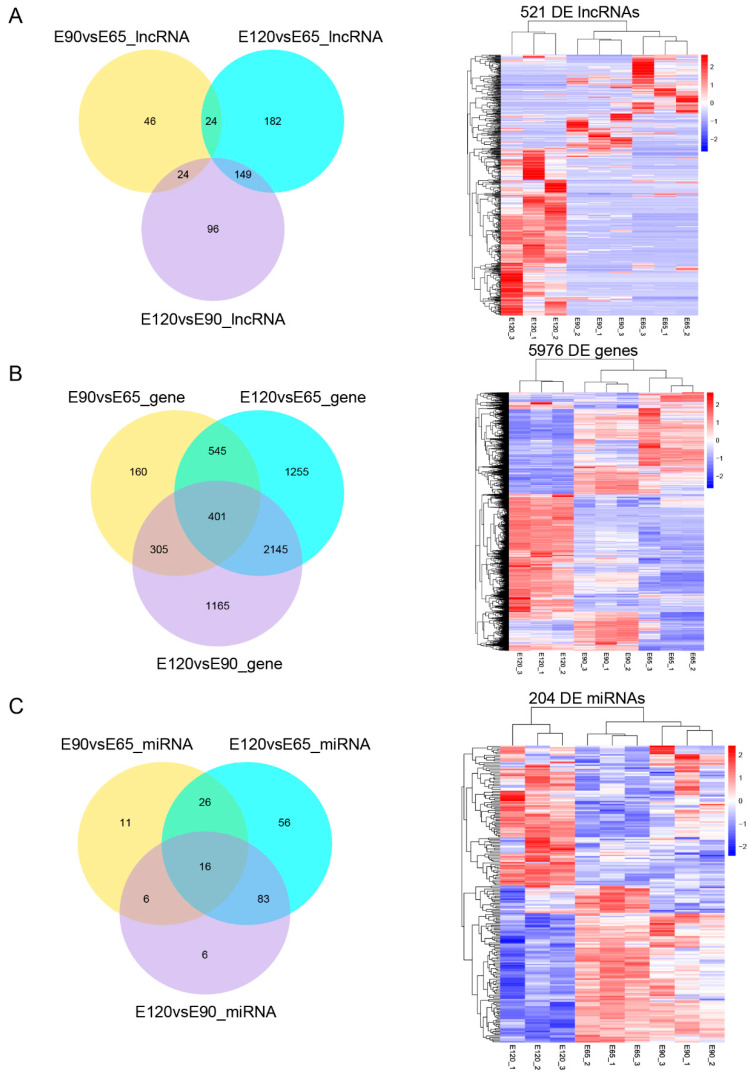
The differentially expressed lncRNAs and genes between E65, E90, and E120 in skin. (**A**) The Venn diagram and heat-map of the differentially expressed lncRNAs. (**B**) The Venn diagram and heat-map of the differentially expressed genes. (**C**) The Venn diagram and heat-map of the differentially expressed miRNAs.

To determine whether ncRNAs are involved in HF morphogenesis, the DE ncRNAs and genes from induction, organogenesis, and cytodifferentiation stages were calculated by FPKM or TPM, and the overlapping DE ncRNAs and genes in both groups were visualized using Venn diagrams (Figure 1, Supplementary Files S6–S8). In addition, up- or down-DE ncRNAs and genes in both groups were visualized using volcano plots (Figure S1). The top 15 up-DE ncRNAs are shown in Table 2, and lncRNA-H19, chi-let-7b-5p, and miR-200b were identified, which were involved in HF development and cycling functions [18,22,23].

To confirm the reliability of the sequencing results, we randomly selected five genes (*KRT23*, *KRT25*, *TGFBR3*, *FZD10*, and *DKK1*), five lncRNAs (H19, TCONS_00462852, TCONS_00021925, XR_001917911.1, and XR_001296756.2), and five miRNAs (miR-184, miR-29a-3p, miR-380-3p, miR-150, and miR195-3p) to validate their expression patterns using RT-qPCR. The results were in concordance with the RNA-seq data, suggesting that the expression patterns based on RNA-seq data were reliable (Figure S2).

LncRNAs can regulate the expression of target genes in cis or trans. The cis actions of lncRNAs are listed in Supplementary File S9. Interestingly, we found that HF development-related genes such as *LEF1*, *HOXC13*, and *LHX2* were located near the TCONS_00208035, TCONS_00158164, and TCONS_00385025 loci, respectively (Supplementary File S11). On the other hand, the trans target genes of lncRNAs are listed in Supplementary File S10. As a result, we detected some important genes that affected HF development, including *DKK1*, *FGF5*, and *Msx2*, that were the targets of H19 (XR_001917728.1), TCONS_00735605, and TCONS_00597833, respectively (Supplementary File S11).

### 2.3. KEGG Pathway Analysis of DE Genes

To explore the key pathways of HF development, the functions of DE genes were predicted by KEGG analysis. The top 20 most enriched pathways are listed in Figure S3. The most enriched pathways between E65 and E90 were extracellular matrix (ECM)–receptor interaction, focal adhesion, and systemic lupus erythematosus (Figure S3A). The most enriched pathways between E90 and E120 were valine, leucine and isoleucine degradation, biosynthesis of amino acids, and ECM–receptor interaction (Figure S3B). Moreover, the most enriched pathways between E65 and E120 were valine, leucine and isoleucine degradation, biosynthesis of amino acids, and alcoholism (Figure S3C). Among the identified KEGG pathways, some belonged to conventional pathways associated with HF development, such as Notch, TGF-beta, and Wnt signaling pathways (Figure S3, Supplementary File S12).

### 2.4. lncRNA–miRNA–mRNA Networks

To explore the molecular mechanisms of ncRNAs involved in HF development, we performed regulatory network analysis of ncRNAs and genes in the three comparison groups. MiRNAs have broad regulatory functions involving RNA silencing and post-transcriptional regulation of gene expression, while lncRNAs also have extensive regulatory functions. We constructed lncRNA–miRNA–gene groups with the lncRNA as the decoy, miRNA as the center, and gene as the target (Figure S4). Among them, we found that lots of keratin family member-encoding genes (*KRT14*, *KRT32*, *KRT74*, *KRT80*, and *KRT82*), a keratin-associated protein gene (*KAP8*), and transcription factors (*Hoxc13*, *LHX2*, and *Foxn1*) were in the regulatory network, such as TCONS_00620110-chi-miR-665-*KRT82*, TCONS_00524724-chi-miR-665-*KAP8*, TCONS_00141916-chi-miR-214-5p-*Hoxc13*, and TCONS_00129107-chi-miR-296-3p-*LHX2*. The results indicated that the expression of genes in the skin during HF development was regulated by a ceRNA regulatory network.

### 2.5. LncRNA H19 Promotes the Proliferation of DP Cells

**Figure 2:**
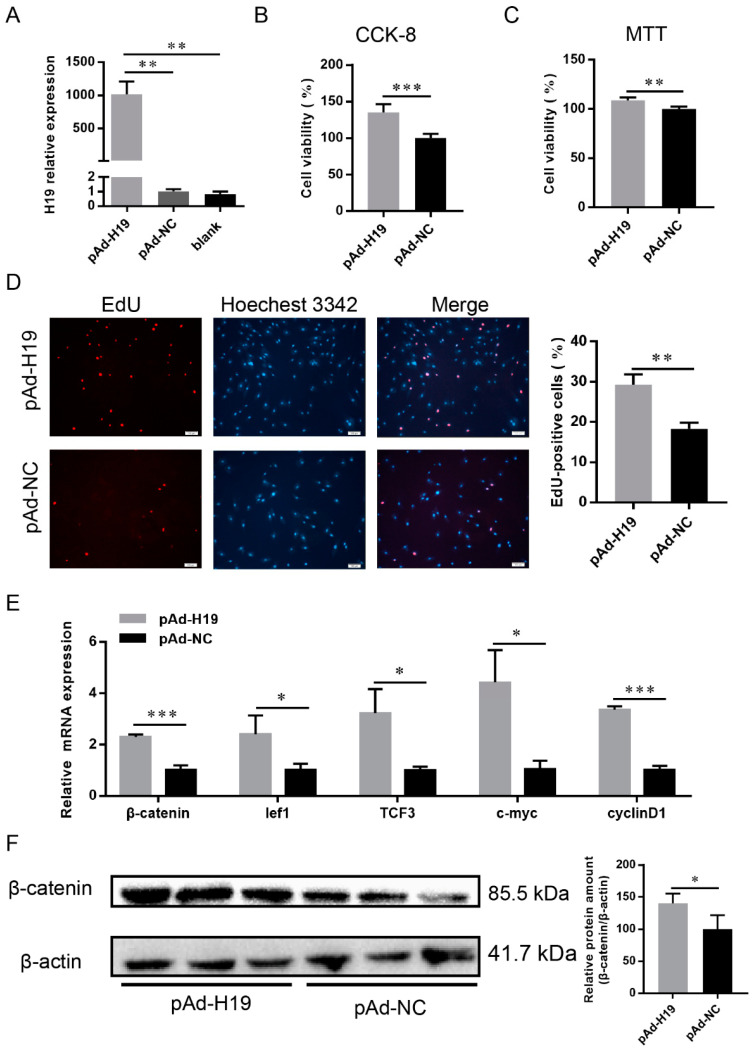
H19 promotes dermal papilla cells proliferation. (**A**) RT-qPCR of H19 expression after transfection with adenoviruses pAd-H19, pAd-NC, and blank control. (**B**) Cell viability was determined by CCK-8 assay. (**C**) Cell viability was determined by MTT assay. (**D**) Cell viability was determined by EdU assay, red represents EdU staining and blue represents cell nuclei stained with Hoechst 33342; the scale bar stands for 100 μm. (**E**) Relative mRNA level of Wnt signaling pathway and downstream factors as detected by RT-qPCR. (**F**) β-catenin protein was detected by Western blot. The data were presented as mean ± SEM for 3 biological replicates, * *p* < 0.05, ** *p* < 0.01, *** *p* < 0.001.

LncRNA H19 was significantly more highly expressed at E65 and E90 than at E120 (Figure S2B), and the ceRNA network indicated that lncRNA H19 could act as a decoy to relieve miRNA-inhibiting effects on genes involved in HF development (Figure S4B); therefore, the effects of lncRNA H19 on the proliferation of DP cells were explored. The overexpressed adenovirus pAd-H19 was transfected into DP cells, and H19 was overexpressed more than 1000-fold over the control (Figure 2A). Cell counting kit-8 (CCK-8) and MTT assays revealed that the overexpression of H19 enhanced the viability of DP cells after 48 h of transfection (Figure 2B,C). Similarly, the cultured DP cells showed significantly more mitotic activity (high percent of EdU-positive cells; Figure 2D).

To explore whether H19 regulated DP cell proliferation by activating the Wnt pathway, pAd-H19 was transfected into DPs. After 48 h, RT-qPCR and Western blot results indicated that the overexpression of H19 promoted the expression of *β-catenin* at mRNA and protein levels (Figure 2E,F). Further, the Wnt pathway downstream factors *lef1*, *tcf3*, and cell cycle-mediated factors *c-myc* and *cyclinD1* were up-regulated (Figure 2E). These findings revealed that H19 promoted the cell cycle by stimulating Wnt pathway activation and might serve as a potential proliferation regulator in DP cells.

### 2.6. LncRNA H19 Functioned as a ceRNA for Chi-miR-214-3p

**Figure 3:**
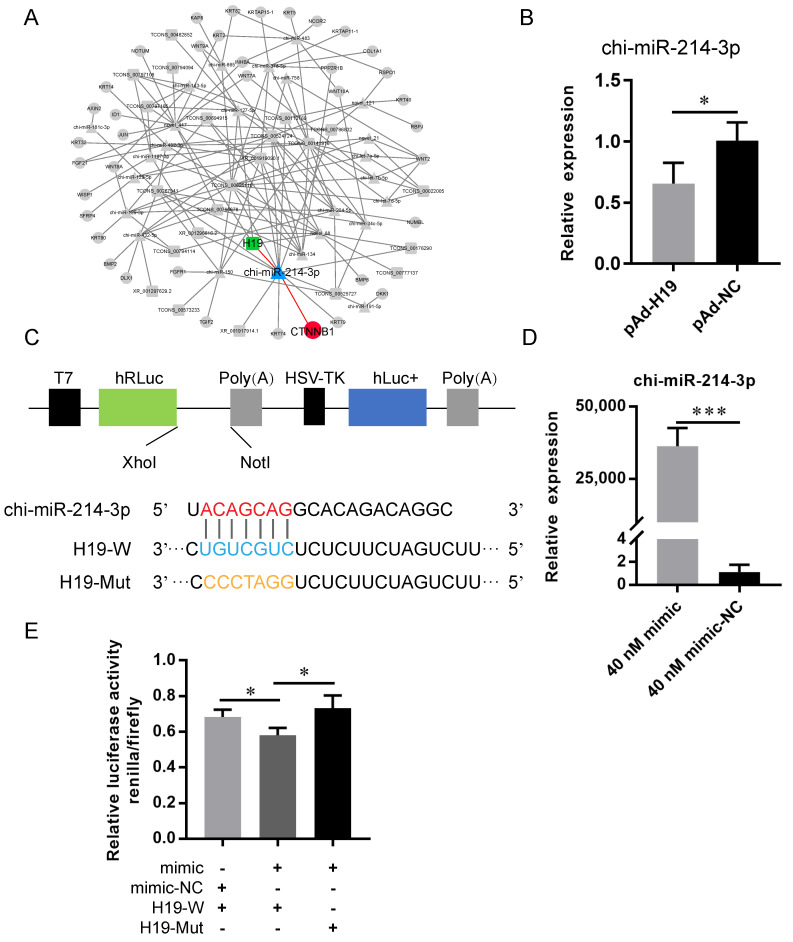
H19 targeted chi-miR-214-3p. (**A**) The ceRNA network showed H19-chi-miR-214-3p-β-catenin. H19 was shown in green, chi-miR-214-3p was shown in blue, and β-catenin (CTNNB1) was shown in red. (**B**) RT-qPCR of chi-miR-214-3p expression after transfection with adenoviruses pAd-H19 and pAd-NC. (**C**) Schematic of the double-luciferase assay vector. Red nucleotide sequences represented the seed sequences of chi-miR-214-3p. The binding site of chi-miR-214-3p in H19 was shown in blue. Orange nucleotide sequences represented the mutation in the seed-binding site of H19. (**D**) RT-qPCR detected the effect of chi-miR-214-3p mimic in HEK293T cells. (**E**) Analysis of the luciferase reporter assay after transfecting with chi-miR-214-3p mimic-NC + H19-W, chi-miR-214-3p mimic + H19-W, or chi-miR-214-3p mimic + H19-Mut. The data were presented as mean ± SEM for 3 biological replicates, * *p* < 0.05. *** *p* < 0.001.

The ceRNA network analysis showed that H19 could act as a chi-miR-214-3p sponge (Figure 3A). Then, the expression level of chi-miR-214-3p was investigated after the overexpression of H19. The RT-qPCR results showed that the expression of chi-miR-214-3p was significantly decreased (*p* < 0.05; Figure 3B). A dual-luciferase assay was performed to validate and explore whether chi-miR-214-3p could bind to the putative binding sites of H19. A 393 bp fragment of H19 containing the chi-miR-214-3p binding site was cloned into the psiCHECK-2 vector to yield psiCHECK-H19-W. A separate fragment containing a 6-base mutation in the seed-binding site of H19 was also moved into the psiCHECK-2 plasmid vector as psiCHECK-H19-Mut (Figure 3C). To overexpress chi-miR-214-3p, its mimic and mimic-NC were transfected into HEK293T cells. The transfection efficiency was detected by RT-qPCR, and the results revealed that the supplementation of 40 nM of chi-miR-214-3p mimic significantly increased the expression level of chi-miR-214-3p compared with the mimic-NC group (Figure 3D). The dual-luciferase assay results revealed that renilla luciferase activity was significantly reduced after the co-transfection of chi-miR-214-3p mimic with psiCHECK-H19-W reporter vector, compared with the corresponding control, while it came to the control level when the putative chi-miR-214-3p binding sites of H19 were mutated (Figure 3E). These results indicated that H19 acted as a ceRNA for chi-miR-214-3p.

### 2.7. Chi-miR-214-3p Suppressed the Proliferation of DP Cells by Targeting β-Catenin

**Figure 4:**
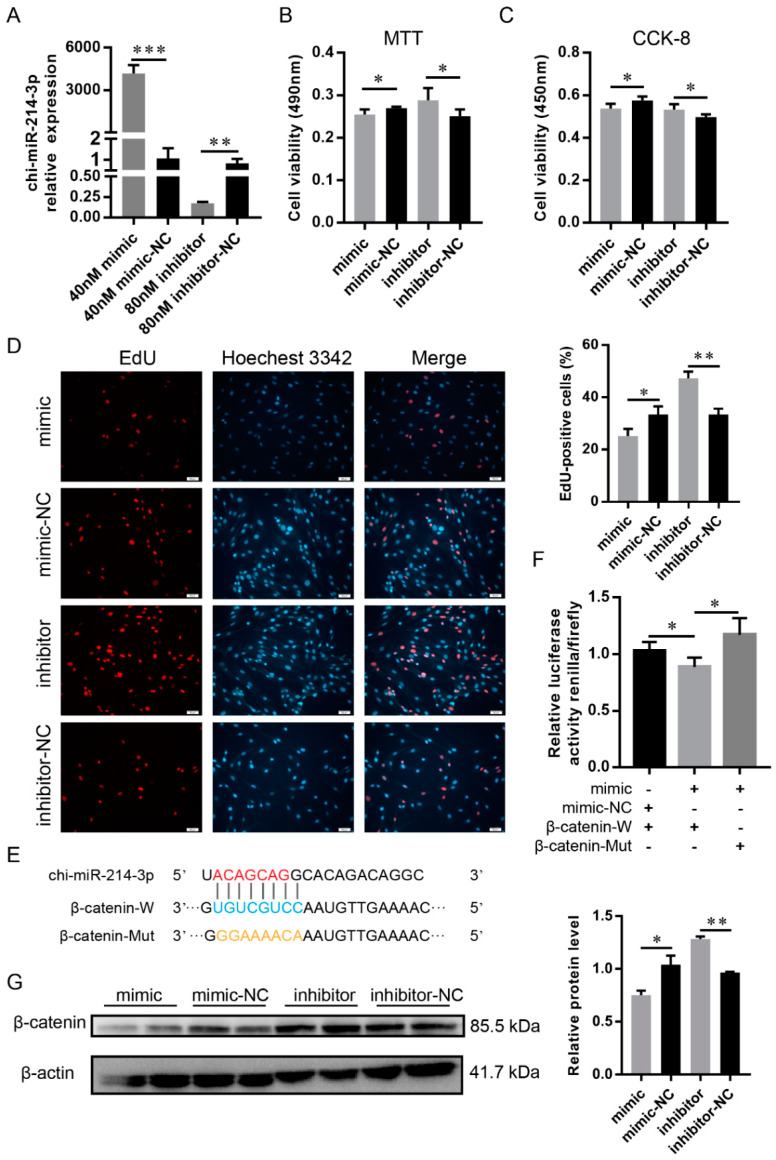
Chi-miR-214-3p targeted β-catenin and inhibited dermal papilla cells proliferation. (**A**) RT-qPCR detected the effect of chi-miR-214-3p mimic and inhibitor. (**B**) MTT analysis of dermal papilla cell proliferation. (**C**) CCK-8 analysis of dermal papilla cell proliferation. (**D**) EdU analysis of dermal papilla cell proliferation; the scale bar stands for 50 μm. Red represents EdU staining and blue represents cell nuclei stained with Hoechst 33342 (**E**) Schematic of the double-luciferase assay vector of β-catenin 3′UTR. Red nucleotide sequences represented the seed sequences of chi-miR-214-3p. The binding site of chi-miR-214-3p in β-catenin 3′UTR was shown in blue. Orange nucleotide sequences represented the mutation in the seed-binding site of β-catenin 3′UTR. (**F**) Analysis of the luciferase reporter assay after transfecting with chi-miR-214-3p mimic-NC + β-catenin-W, chi-miR-214-3p mimic + β-catenin-W, or chi-miR-214-3p mimic + β-catenin-Mut. (**G**) β-catenin protein was detected by Western blot. The data were presented as mean ± SEM for 3 biological replicates, * *p* < 0.05, ** *p* < 0.01. *** *p* < 0.001.

In order to assess the impact of chi-miR-214-3p on the proliferation of DP cells, its mimic and inhibitor were transfected into DP cells. The RT-qPCR analysis revealed that the supplementation of 40 nM of chi-miR-214-3p mimic or 80 nM of chi-miR-214-3p inhibitor significantly increased or decreased the expression level of chi-miR-214-3p compared with their NC groups (Figure 4A). Cell viability was significantly reduced in DP cells with the transfection of chi-miR-214-3p mimic compared with the control group by MTT and CCK-8 assays (*p* < 0.05), whereas the cell viability was significantly increased with the supplementation of chi-miR-214-3p inhibitor compared to its control (*p* < 0.05; Figure 4B,C). Similarly, the cultured DP cells had significantly less or more mitotic activity with chi-miR-214-3p overexpression or suppression (Figure 4D). Combined, the above-mentioned results confirmed that chi-miR-214-3p inhibited the proliferation of DP cells.

The ceRNA network analysis showed that chi-miR-214-3p could target *β-catenin* (Figure 3A). In order to explore whether chi-miR-214-3p regulated the proliferation of DP cells by *β-catenin*, a dual-luciferase assay, RT-qPCR, and Western blot analysis were performed. Renilla luciferase activity was significantly reduced after the co-transfection of wild-type psiCHECK-β-catenin-W vector and miR-214-3p mimic, compared with the corresponding control and mutant-type psiCHECK-β-catenin-Mut vector (Figure 4E,F). The mRNA expression level of *β-catenin* was not significantly changed with the overexpression or inhibition of chi-miR-214-3p in goat dermal papilla cells (Figure S5). Western blot results indicated that the overexpression of miR-214-3p suppressed the expression of β-catenin, whereas the inhibition of chi-miR-214-3p resulted in the reverse (Figure 4G). Thus, it could be deduced that chi-miR-214-3p directly targeted the 3′UTR of *β-catenin* to inhibit its translation in DP cells.

### 2.8. β-Catenin Promoted the Proliferation of DP Cells

**Figure 5:**
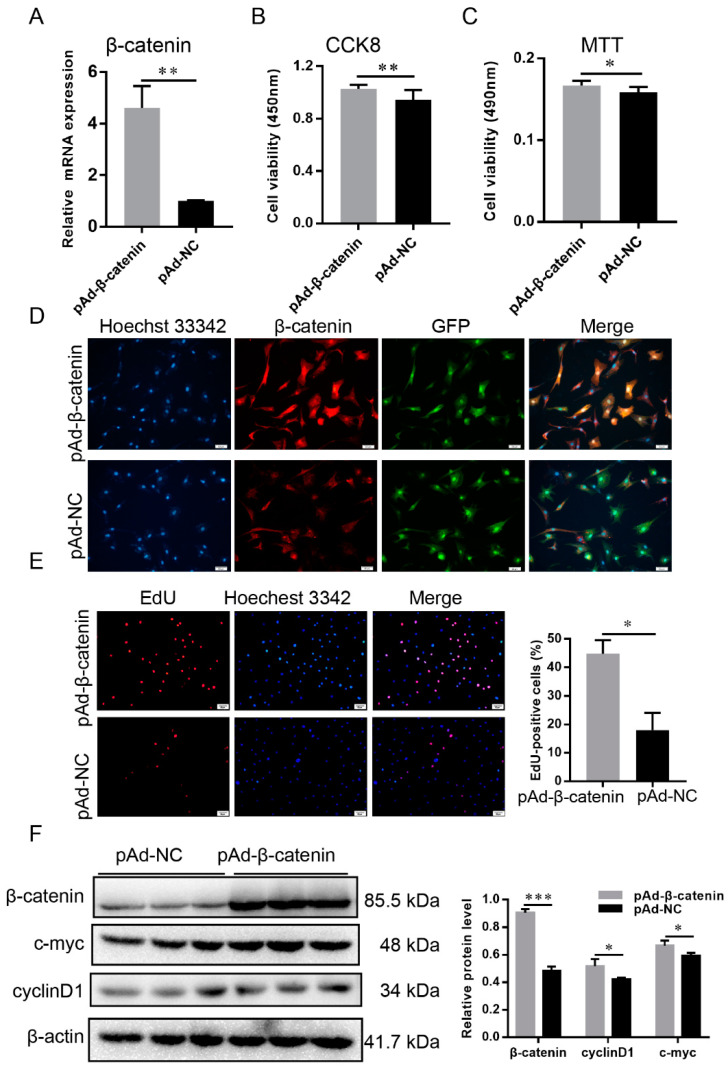
β-catenin promoted dermal papilla cell proliferation. (**A**) RT-qPCR detected the effect of pAd-β-catenin adenovirus. (**B**) CCK-8 analysis of dermal papilla cell proliferation. (**C**) MTT analysis of dermal papilla cell proliferation. (**D**) β-catenin marked by immunofluorescence in dermal papilla cells. Red represents β-catenin staining, green represents the transduction efficiency of recombinant adenoviruses, and blue represents cell nuclei stained with Hoechst 33342. The scale bar stands for 50 μm. (**E**) EdU analysis of dermal papilla cell proliferation; the scale bar stands for 50 μm. Red represents EdU staining and blue represents cell nuclei stained with Hoechst 33342. (**F**) β-catenin, c-myc, and cyclinD1 protein were detected by Western blot. The data were presented as mean ± SEM for 3 biological replicates, * *p* < 0.05, ** *p* < 0.01, *** *p* < 0.001.

In order to understand the role of *β-catenin* in the proliferation of DP cells, the overexpressed adenovirus pAd-β-catenin was transfected into DP cells. RT-qPCR and Western blot results revealed that β-catenin was significantly increased compared with pAd-NC (Figure 5A,F). From the immunofluorescence result, β-catenin was assembled in nuclei after 48 h of transfection (Figure 5D). The viability of DP cells had been enhanced by CCK-8 and MTT assays (Figure 5B,C). Similarly, the cultured DP cells showed significantly greater mitotic activity (Figure 5E). Western blot results revealed that the expressions of cell cycle-mediated factors, c-myc and cyclinD1, were improved (Figure 5F). These findings revealed that *β-catenin* promoted the cell cycle of DP cells.

### 2.9. LncRNA H19 Promoted Dermal Papilla Cell Proliferation through the Chi-miR-214-3p/β-Catenin Axis

**Figure 6:**
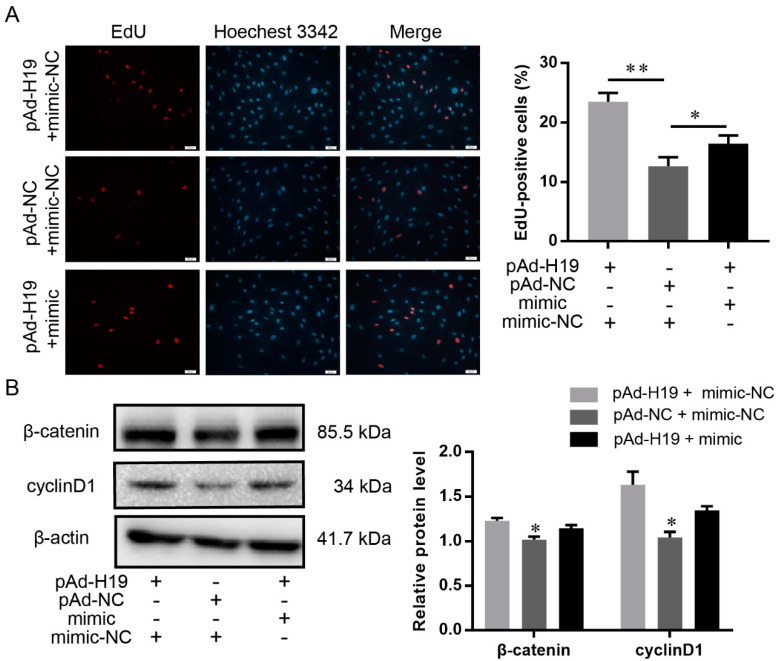
chi-miR-214-3p weakened the positive effect of H19 on dermal papilla cell proliferation. (**A**) EdU analysis of dermal papilla cell proliferation after transfecting with pAd-H19 + chi-miR-214-3p mimic-NC, pAd-NC + chi-miR-214-3p mimic-NC, or pAd-H19 + chi-miR-214-3p mimic. The scale bar stands for 50 μm. Red represents EdU staining and blue represents cell nuclei stained with Hoechst 33342. (**B**) β-catenin and cyclinD1 protein were detected by Western blot. The data were presented as mean ± SEM for 3 biological replicates, * *p* < 0.05, ** *p* < 0.01.

To confirm that H19 acted as a chi-miR-214-3p sponge to mediate *β-catenin*, pAd-H19 was co-transfected with chi-miR-214-3p mimic or mimic-NC into DP cells. The cultured DP cells had significantly greater mitotic activity after the co-transfection of pAd-H19 and mimic-NC, compared with pAd-NC+ mimic-NC, whereas the co-transfection of pAd-H19 and chi-miR-214-3p mimic decreased this effect (Figure 6A).

We then detected the protein expression of β-catenin and cell cycle-mediated factor cyclinD1. As expected, H19 significantly increased the expression of β-catenin and cyclinD1, while this effect was inhibited by chi-miR-214-3p overexpression (Figure 6B). Together, these findings indicated that H19, by binding chi-miR-214-3p, acted as a decoy to relieve miRNA-inhibiting effects on β-catenin.

## 3. Discussion

Skin is composed of multiple different cell types (melanocytes, adipocytes, and fibroblasts) and accessory organs (HFs and sweat glands); it is the first line of defense and covers the entire body [24]. HFs originate from the interaction between the epidermis and dermis at the embryonic stage, including primary HFs and SHFs. The number and density of embryonic SHFs directly determine the yield and diameter of cashmere fibers.

In recent years, increasing evidence has demonstrated that ncRNAs play important roles in the development of HF cycling in cashmere goats [20,21,25,26]. However, very few studies have been conducted regarding the potential role of ncRNAs in goat skin’s fetal HF development [27,28]. In the present study, we systematically identified the ncRNAs and mRNAs involved in cashmere goat skin during three different fetal HF developmental stages using RNA-sequencing technology. We subsequently characterized putative lncRNAs and miRNAs to elucidate their diverse features in order to provide an insight into their relationship with HF development of the cashmere goat. In addition, we identified a total of 10 485, 605, and 40 639 expressed lncRNAs, miRNAs, and mRNAs, respectively, during goat HF morphogenesis, including many lncRNAs that have never been reported in previous data.

### 3.1. Regulation of HF Development

Fetal HF development has been traditionally subdivided into three main stages: induction, organogenesis, and cytodifferentiation [29]. Several signaling pathways, including Wnt, Eda, Notch, and BMP signaling, have been established as critical for HF development in mice [30,31,32]. Correspondingly, some conventional pathways were also found in the current study, including Notch, TGF-beta, and Wnt signaling pathways. At the same time, more transcription factors are being shown to be involved in HF differentiation and hair shaft growth. GATA3 is specifically expressed in precursor cells of the inner root sheath (IRS) [33]; the IRS structure cannot be formed during hair follicle formation, resulting in abnormal hair structure when *Gata3* is specifically knocked out [34]. Research shows that Foxq1 plays an important role in the medullary growth of mouse hair shafts [35,36], and Msx2 and Hoxc13 have important roles in HF differentiation during hair shaft growth [37,38]. Consistent with these results, we found that GATA3, Foxq1, Msx2, and Hocx13 were highly expressed in E120, which indicated that these transcription factors play an important role in the differentiation of cashmere goat HFs.

### 3.2. LncRNAs and miRNAs Play an Important Role in HF Development

Multiple studies have shown that lncRNAs play an indispensable role in HF development [18,39]. In this study, we identified 521 DE lncRNAs in total, some of which were adjacent or co-expressed with HF development-related genes, which indicated their possible functions in HF development. These lncRNAs should be further explored in order to serve as important candidate markers for precision breeding. Zhu et al. used network analysis to show that H19 may regulate HF cycles, which were more highly expressed at anagen than at either telogen or catagen phases in Liaoning Cashmere goats [40]. Here we found that lncRNA H19 was more highly expressed in both induction and organogenesis than in the cytodifferentiation stage; subsequent functional assays showed that it could promote DP cell proliferation through activating the Wnt signaling pathway.

Although previous research in humans or mice has identified a few miRNAs and revealed their function in HF morphogenesis and development [15,16,23], until now, little was known about cashmere goats, specifically cashmere follicle initiation and development. In this study, chi-miR-22-5p and chi-miR-200a/b were more highly up-regulated in cytodifferentiation than in induction and organogenesis stages; however, chi-miR-200c and chi-miR-214-3p/5p were more highly up-regulated in induction than in organogenesis or cytodifferentiation stages, indicating that they may have different roles in different developmental stages. Target prediction showed that some differentially expressed miRNAs target important genes involved in HF development and cycling. Furthermore, functional assays showed that chi-miR-214-3p could inhibit DP cell proliferation through suppressing the Wnt signaling pathway, which was opposite to H19.

### 3.3. LncRNA Could Function as a ceRNA during HF Development

Salmena et al. proposed a competitive endogenous RNA hypothesis in 2011 [41]. This hypothesis considered that miRNAs were the core elements in the ceRNA network (mRNA, lncRNA, circRNA (circular RNA), or pseudogene as ceRNA), which competed with one or more miRNA response elements to regulate the function of other RNAs. Studies have shown that lncRNAs are involved in the HF cycle of cashmere goats as ceRNA [20,21], but until now, little was known about cashmere HF initiation and development in the fetal stages. Here, we constructed ceRNA networks jointed by lncRNAs, miRNAs, and mRNAs in fetal HF developmental stages. Furthermore, we found that some keratin family member-encoding genes (*KRT14*, *KRT32*, *KRT74*, *KRT80*, and *KRT82*), a keratin-associated protein gene (*KAP8*), and transcription factors (*Hoxc13*, *LHX2*, and *Foxn1*) were in the regulatory network, which indicated that lncRNAs harbor potential miRNA recognition elements and participate in a complex ceRNA network in hair growth and development. The network brings to light an unknown miRNA regulatory network in cashmere induction and development. It also suggests that lncRNAs may play crucial roles in cashmere development in fetal stages.

In particular, the ceRNA network showed that lncRNA H19 could act as a chi-miR-214-3p sponge to regulate the expression of β-catenin. Subsequently, the dual-luciferase assay, RT-qPCR, and Western blot analysis were performed to validate and explore whether chi-miR-214-3p could bind to the putative binding sites of H19 and β-catenin; this was in accordance with the previous study that found that miR-214-3p directly targets the 3′UTR of *β-catenin* to inhibit its protein expression in mice [15]. The results indicated that chi-miR-214-3p could bind to the putative binding sites of H19 and β-catenin. Subsequently, the *β-catenin* functional assays and chi-miR-214-3p recovery assay indicated that lncRNA H19, by binding miR-214-3p, acts as a decoy to relieve the chi-miR-214-3p-inhibiting effect on β-catenin. It needs to be further tested at an individual level to verify whether it can promote the improvement of cashmere yield and quality, which can be considered as an important candidate marker for cashmere goat molecular breeding.

## 4. Materials and Methods

### 4.1. Animals and Samples

All the experimental goats were obtained from the Shanbei Cashmere Goat Engineering Technology Research Center of Shaanxi province in China and were fed with the local cashmere goat standard feed (DB61/T583-2013). All pregnant goats were prepared using artificial insemination and all of the experimental procedures involving goats in this study were approved by the Experimental Animal Manage Committee of Northwest A&F University (Approval ID: 2014ZX08008–002).

In order to deeply understand the molecular profiles during the morphogenesis of cashmere goat HFs, we collected nine skin samples of E65-, E90-, and E120-stage fetuses (three fetuses in each stage). These time points were determined according to previous studies [3,4,5], which correspond to HF induction, organogenesis, and cytodifferentiation stages. Nine goat fetuses were isolated using cesarean section when the pregnant goats were anesthetized with the compound ketamine. Samples of goat fetus back skin (1 cm^2^, 3 mm deep) were immediately transferred to an RNA sample protector (Takara, Dalian, China) and stored at −80 °C for future analysis.

### 4.2. Transcriptome Sequencing and Bioinformatics Analysis

First, the collected skin tissues from nine fetuses were ground in liquid nitrogen, then total RNA was extracted using Trizol reagent (Invitrogen, Waltham, CA, USA) following the manufacturer’s instructions. Two libraries were constructed from each fetus sample: a lncRNA library and a miRNA library. RNA-seq and subsequent bioinformatics analyses were performed as previously described [20]. Fragments per kb per million reads (FPKM) of both lncRNAs and coding genes were calculated by Cuffdiff (v2.1.1) in each sample. MiRNA expression levels were estimated by TPM (transcript per million) with the following criteria: normalization formula: normalized expression = actual miRNA count/total count of clean reads × 1,000,000. Sample quality was assessed on the Agilent Bioanalyzer 2100 system (Agilent Technologies, Santa Clara, CA, USA). The libraries were sequenced on an Illumina HiSeq 4000 platform (Illumina, CA, USA) as 150 bp paired-end reads. Small RNA libraries were sequenced on an Illumina HiSeq 2500 platform (Illumina, CA, USA) using 50 bp single-end reads.

### 4.3. Validation of Gene Expression by Real-Time Quantitative PCR (RT-qPCR) Analysis

The total RNAs used for RT-qPCR analysis were the same as those for RNA-seq. For lncRNAs and mRNAs, the first-strand cDNA was obtained using a PrimeScript™ RT reagent Kit with gDNA Eraser (Takara, Dalian, China). For miRNA quantification, the first-strand cDNA was obtained using the aforementioned kit with a specific stem-loop primer of each miRNA and universal U6 reverse primer. Then, they were subjected to the quantification of mRNAs and ncRNAs using TB GreenTM Premix Ex TaqTM II (Takara, Dalian, China) on a Roche LightCycler^®^ 96 System with β-actin or U6 as endogenous controls. The RT-qPCR procedure was as follows: 95 °C for 1 min, followed by 40 cycles of 95 °C for 10 s, the optimized annealing temperature for 30 s, and extension at 72 °C for 30 s.

The primers used for this experiment are listed in Supplementary File S1. Each stage (E65, E90, and E120) included at least three samples, and all of the reactions were performed in triplicate for each sample. Gene expression was quantified relative to endogenous gene expression using the 2^−^^△△CT^ method through LightCycler^®^ 96 and Microsoft Excel 2019. Student’s *t*-test was used for statistical analysis, and a *p*-value < 0.05 was considered significant. Values are expressed as means ± SD.

### 4.4. KEGG Pathway and lncRNA Target Analysis

ClusterProfiler R package were used to test the statistical enrichment of DE genes in KEGG pathways; KOBAS 3.0 (http://kobas.cbi.pku.edu.cn/index.php, accessed on 10 August 2020) was used to investigate the statistical enrichment of candidate targets in KEGG pathways. Co-expression analysis was based on calculating the Pearson correlation coefficients between coding genes and non-coding transcripts according to their expression levels. An absolute value of the parameter PCC ≥ 0.95, *p*-value < 0.01, and false discovery rate (FDR) < 0.01 were used for identifying genes for further analysis. For identifying cis-regulation, lncRNAs that act on neighboring target genes were investigated. The coding genes 10 k/100 k upstream and downstream of each lncRNA were screened and then analyzed. For identifying trans-regulation, lncRNAs and target genes were identified based on their expression levels. Custom scripts were used to calculate the expression level correlation between lncRNAs and coding genes.

### 4.5. Competing Endogenous RNA (CeRNA) Network Analysis

To reveal the roles and interactions of ncRNAs and mRNAs during HF development, we constructed ncRNA regulatory networks using the DE lncRNA, miRNA, and gene data. Potential miRNA response elements were searched for in the sequences of lncRNAs and genes, and the overlaps between the seed sequence of predicted miRNAs in the binding sites of target genes and lncRNA binding sites were identified as part of the lncRNA–miRNA–gene interaction. The miRNA binding sites were predicted by miRBase (http://mirbase.org, accessed on 15 August 2020), the lncRNA–miRNA interactions were predicted by miRanda (http://miranda.org.uk/, accessed on 15 August 2020), while the miRNA–gene interactions were predicted by miRanda and RNAhybrid (https://directory.fsf.org/wiki/RNAhybrid, accessed on 15 August 2020). The interaction network was built and visually displayed using Cytoscape (v3.8.2) software based on the screening of lncRNA–miRNA–gene pairs.

### 4.6. Vector Construction

For the purpose of overexpressing H19 on DP cells, we used the AdEasy system. The H19 sequence was amplified by PCR using goat genomic DNA as a template with the forward primer H19-ALL-F and reverse primer H19-ALL-R. The PCR products were cloned into the pAdTrack-GOI shuttle vector using *Kpn* I and *Not* I enzymes (NEB, Ipswich, MA, USA). Then, the resultant plasmid was linearized by *Pme* I (NEB, MA, USA) and transformed into BJ5183 AdEasier cells for recombination. The confirmed recombinant adenovirus plasmids were digested with *Pac* I (NEB, MA, USA) and transfected into HEK-293A cells for generating adenoviruses pAd-H19 within 14-20 days. In order to overexpress β-catenin, the full-length ORF of β-catenin was amplified by PCR using goat skin cDNA as a template with the forward primer CTNNB1-CDS-F and reverse primer CTNNB1-CDS-F. Then, the adenoviruses’ plasmids and adenovirus pAd-β-catenin were generated as described above. The pAdTrack-GOI plasmids were used to generate the control adenovirus pAd-NC.

Chi-miR-214-3p mimic, inhibitor, and their corresponding negative control oligonucleotides were synthesized by Shanghai GenePharma Co., Ltd. (Shanghai, China). Mimic and mimic-NC were double-stranded RNAs; inhibitor and inhibitor-NC were single-stranded RNAs. The mimic sequence was UACAGCAGGCACAGACAGGC, the mimic-NC sequence was UUGUACUACACAAAAGUACUG, the inhibitor sequence was GCCUGUCUGUGCCUGCUGUA, and the inhibitor-NC sequence was CAGUACUUUUGUGUAGUACAA. The chi-miR-214-3p mimic, inhibitor, and their corresponding negative control were transfected into dermal papilla cells using Lipofectamine 2000 (Invitrogen, Carlsbad, CA, USA) according the manufacturer’s protocol.

In order to explore whether chi-miR-214-3p could bind to the putative binding sites of *H19* and *β-catenin*. The H19 and β-catenin-3′UTR sequences including the chi-miR-214-3p binding site were amplified by PCR using goat skin cDNA as a template with H19-(mi214)-W-F and H19-(mi214)-W-R, and CTNNB1-W-UTR-F and CTNNB1-W-UTR-R, respectively. Then, the amplified fragments were cloned into psiCHECK-2 dual-luciferase reporter vectors (Promega, St. Louis, MO, USA) using *Xho* I and *Not* I enzymes (NEB, MA, USA). The vectors were named psiCHECK-H19-W and psiCHECK-β-catenin-W. Separately, the psiCHECK-H19-Mut and psiCHECK-β-catenin-Mut vectors were generated with a several-bases mutagen in the chi-miR-214-3p binding site by two pairs of mutagenic primers H19-(mi214)-Mut-F and H19-(mi214)-Mut-R, and CTNNB1-W-UTR-F and CTNNB1 Mut-UTR-R, respectively. All the primers used for vector construction are listed in Supplementary File S1. The constructed vectors were further verified by sequencing.

### 4.7. MTT Assay

The DP cells were seeded in 96-well plates with 1 × 10^4^ cells per well for 48 h after the transfection of chi-miR-214-3p mimic, chi-miR-214-3p inhibitor, adenovirus pAd-H19, or adenovirus pAd-β-catenin. 50 μL of MTT solution [3-(4, 5-dimethylthiazol-2-yl)-2, 5-diphenyltetrazolium bromide, 5 mg/mL] (Sigma, St. Louis, MO, USA) was added to each well and the cells were incubated at 37 °C for 4 h in the dark. Then, 150 μL of DMSO (Solarbio, Beijing, China) was added to dissolve the precipitate. Absorbance values were determined at 490 nm using a SynergyH1 multi-detector microplate reader (BioTek, Winooski, VT, USA).

### 4.8. CCK-8 Assay

The cell proliferation was also assessed by a CCK-8 assay. After 48 h of transfection, 10 μL of CCK-8 reagent (Sangon Biotech, Shanghai, China) was added to each well and incubation was continued for 1 h. The absorbance value of all samples was detected using the SynergyH1 multi-detector microplate reader (BioTek, Winooski, VT, USA) at 450 nm.

### 4.9. EdU Assay

The cell proliferation was also evaluated using the Cell-Light EdU Apollo567 In Vitro Kit (100T) (RiboBio, Guangzhou, China). The cultured medium was aspirated after 48 h of transfection and incubated with EdU medium for 6 h, and the EdU-positive cells were detected following the manufacturer’s protocol.

### 4.10. Dual-Luciferase Assay

The chi-miR-214-3p mimic and corresponding negative controls were co-transfected into HEK293T cells with psiCHECK-H19-W, psiCHECK-β-catenin-W, psiCHECK-H19-Mut, or psiCHECK-β-catenin-Mut vectors when the cells were about 80% confluence in 24-well plates by Lipofectamine 2000. The protocol of dual-luciferase assays was described in a previous study [42].

### 4.11. Western Blot

The process of total protein extraction and protocol of Western blot were described in a previous study [43]. The antibody information was as follows: anti-β-catenin (dilution ratio 1:5000, Proteintech, Wuhan, China, Cat. No.:51067-2-AP), anti-β-actin (dilution ratio 1:2000, Proteintech, Wuhan, China, Cat. No.: HRP-60008), anti-c-myc (dilution ratio 1:1000, BD Biosciences, NY, USA, Cat. No.: 551101), anti-cyclinD1 (1:5000, Proteintech, Wuhan, China, Cat. No.: 60186-1-Ig), secondary antibodies of anti-immune rabbit IgG–HRP (dilution ratio 1:5000, Sangon Biotech, Shanghai, China, Cat. No.: D1110058) and anti-immune mouse IgG–HRP (dilution ratio 1:5000, Sangon Biotech, Shanghai, China, Cat. No.: D1110087).

### 4.12. Statistical Analysis

Histograms in this research were analyzed by GraphPad Prism 7.0 software (GraphPad Software, San Diego, CA, USA) with a *t*-test. Statistical significance was considered as * *p* < 0.05, ** *p* < 0.01, and *** *p* < 0.001. Image J was handled for gray value analysis of gels.

## 5. Conclusions

**Figure 7:**
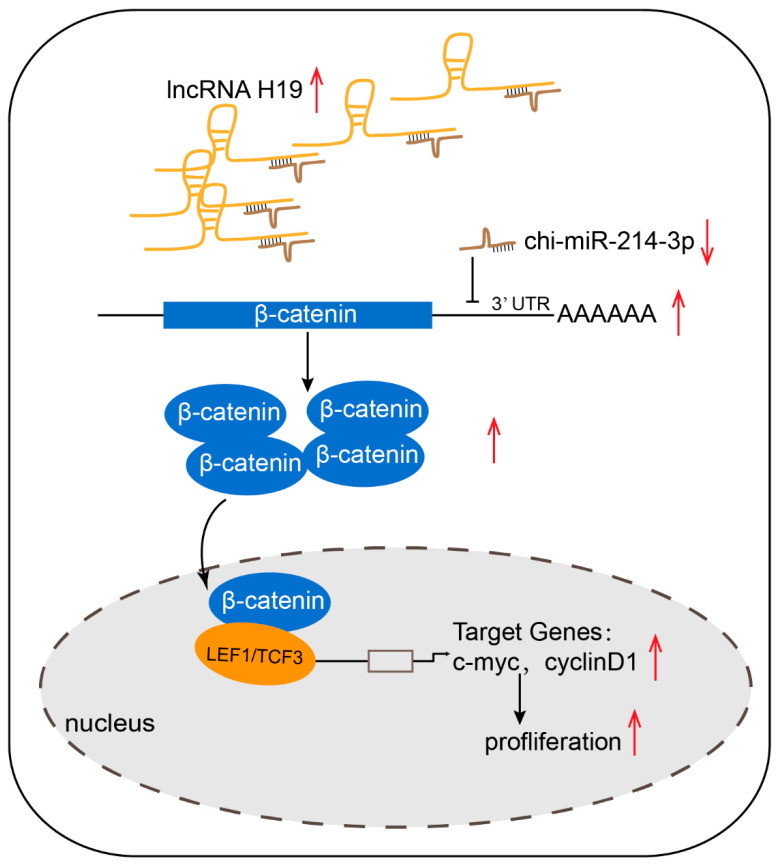
Model of H19 regulating the proliferation of cashmere goat dermal papilla cells. Red arrow up means increase, arrow down means decrease.

In summary, we systematically identified the lncRNAs, miRNAs, and mRNAs involved in cashmere goat skin during three different fetal HF development stages using RNA-sequencing technology. A total of 521 DE lncRNAs, 204 DE miRNAs, and 5976 DE genes were identified. Then, integrative analyses of the lncRNAs, miRNAs, and mRNAs were performed to determine their predicted roles and regulatory relationships in HF development. Furthermore, we found that lncRNA H19 promoted DP cell proliferation through the chi-miR-214/β-catenin axis (Figure 7), which provided new insight into the molecular mechanisms of cashmere development in fetal stages and contributed to the annotation of the goat genome.

## Figures and Tables

**Table 1:** Summary of identified genes and ncRNAs.

	Known	Novel	Known Different	Novel Different
mRNAs	40,364	275	5940	36
lncRNAs	2227	8258	91	430
miRNAs	422	183	180	24

**Table 2:** The information of top 15 up-DE ncRNAs.

Transcript_id	E65_FPKM/TPM	E90_FPKM/TPM	E120_FPKM/TPM
**lncRNAs**			
H19	843.0740	941.2135	114.6905
TCONS_00573233	508.9460	37.8758	380.9373
XR_001296756.2	0.4040	4.7004	133.6244
TCONS_00790678	23.2770	0.0000	99.5389
TCONS_00796832	1.0006	101.5070	1.8325
TCONS_00794094	12.0705	0.0000	76.5603
TCONS_00267940	0.2418	82.8900	0.9865
TCONS_00799045	5.7793	0.0000	37.3490
TCONS_00021925	0.0205	0.0074	30.0650
TCONS_00267941	0.0122	27.4923	0.0000
TCONS_00462852	0.2009	0.6194	24.8990
XR_001297629.2	0.0161	0.0000	23.0225
TCONS_00579021	15.4707	0.1352	4.9993
XR_001917911.1	0.0000	0.6697	16.3470
TCONS_00794114	0.0000	15.9658	0.0000
**miRNAs**			
chi-miR-143-3p	67,656.2395	53,405.3408	143,004.1167
chi-miR-26a-5p	56,081.5294	69,262.7051	71,726.3097
chi-miR-27b-3p	18,121.1064	31,704.0191	48,005.4398
novel_1	9700.2197	21,113.9625	51,116.4677
chi-let-7f-5p	18,722.4699	24,936.8240	29,656.2387
chi-miR-100-5p	34,055.8992	17,383.0249	13,167.1150
chi-miR-411a-5p	27,666.1047	25,927.8426	9216.2797
chi-let-7g-5p	15,255.1331	17,077.0222	21,381.1897
chi-let-7a-5p	9843.2445	13,049.8691	22,008.5678
chi-miR-126-3p	13,413.4404	10,380.2313	19,570.4819
chi-miR-379-5p	16,120.5727	13,927.9794	6478.6473
chi-miR-24-3p	6192.5776	8893.4012	13,070.6241
chi-miR-378-3p	14,459.7160	7918.5068	4337.4918
chi-let-7b-5p	3520.0832	6329.7777	11,805.9702
chi-miR-200b	4858.8871	5182.6072	11,212.8627

## Data Availability

The lncRNA-seq data used in this research are available in the NBCI SRA repository. BioProject accession: PRJNA597198. BioSamples: SAMN13669153, SAMN13669154, SAMN13669155, SAMN13669156, SAMN13669157, SAMN13669158, SAMN26558928, SAMN26558929, and SAMN26558930. The miRNA-seq data used in this research are available in the NBCI SRA repository. BioProject accession: PRJNA814621. BioSamples: SAMN26561582, SAMN26561583, SAMN26561584, SAMN26561585, SAMN26561586, SAMN26561587, SAMN26561588, SAMN26561589, and SAMN26561590.

## Supplementary Materials

The following supporting information can be downloaded at: https://www.mdpi.com/article/10.3390/ijms231710006/s1.
