# Supplementary material for: Comprehensive Transcriptome Analysis of Hair Follicle Morphogenesis Reveals That lncRNA-H19 Promotes Dermal Papilla Cell Proliferation through the Chi-miR-214-3p/β-Catenin Axis in Cashmere Goats
Source: Int J Mol Sci. 2022 Sep 2;23(17):10006. doi: 10.3390/ijms231710006 (PMC9456307; doi:10.3390/ijms231710006)
Supplement: Supplementary file 1 [file ijms-23-10006-s001.zip › ijms-1812373-supplementary/Fig.S3.pdf]

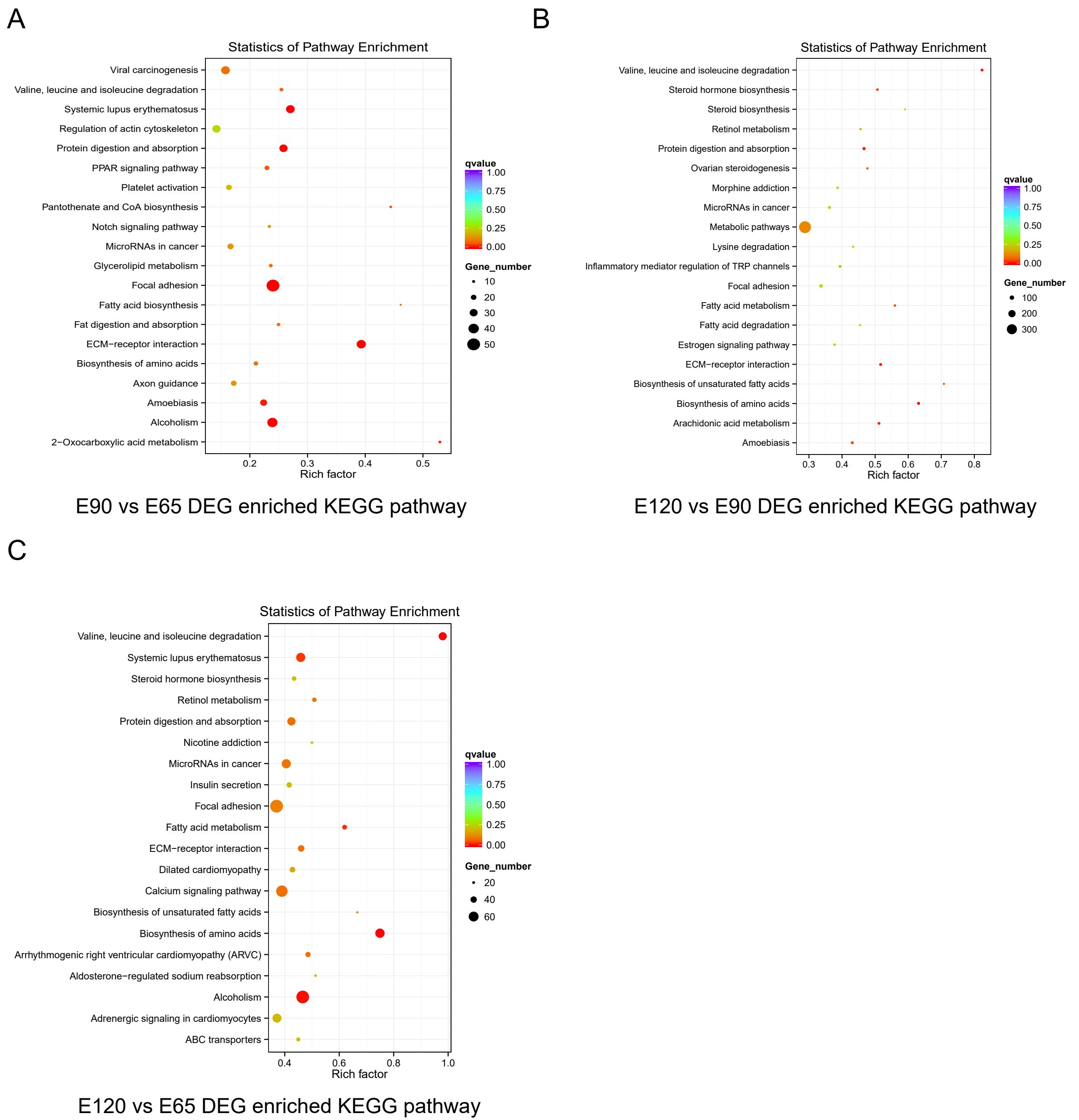
A
Statistics of Pathway Enrichment
Viral carcinogenesis
Valine, leucine and isoleucine degradation
Systemic lupus erythematosus
Regulation of actin cytoskeleton
Protein digestion and absorption
PPAR signaling pathway
Platelet activation
Pantothenate and CoA biosynthesis
Notch signaling pathway
MicroRNAs in cancer
Glycerolipid metabolism
Focal adhesion
Fatty acid biosynthesis
Fat digestion and absorption
ECM−receptor interaction
Biosynthesis of amino acids
Axon guidance
Amoebiasis
Alcoholism
2−Oxocarboxylic acid metabolism
0.2
0.3
0.4
0.5
Rich factor
qvalue
1.00
0.75
0.50
0.25
0.00
Gene_number
10
20
30
40
50
E90 vs E65 DEG enriched KEGG pathway
B
Statistics of Pathway Enrichment
Valine, leucine and isoleucine degradation
Steroid hormone biosynthesis
Steroid biosynthesis
Retinol metabolism
Protein digestion and absorption
Ovarian steroidogenesis
Morphine addiction
MicroRNAs in cancer
Metabolic pathways
Lysine degradation
Inflammatory mediator regulation of TRP channels
Focal adhesion
Fatty acid metabolism
Fatty acid degradation
Estrogen signaling pathway
ECM−receptor interaction
Biosynthesis of unsaturated fatty acids
Biosynthesis of amino acids
Arachidonic acid metabolism
Amoebiasis
0.3
0.4
0.5
0.6
0.7
0.8
Rich factor
qvalue
1.00
0.75
0.50
0.25
0.00
Gene_number
100
200
300
E120 vs E90 DEG enriched KEGG pathway
C
Statistics of Pathway Enrichment
Valine, leucine and isoleucine degradation
Systemic lupus erythematosus
Steroid hormone biosynthesis
Retinol metabolism
Protein digestion and absorption
Nicotine addiction
MicroRNAs in cancer
Insulin secretion
Focal adhesion
Fatty acid metabolism
ECM−receptor interaction
Dilated cardiomyopathy
Calcium signaling pathway
Biosynthesis of unsaturated fatty acids
Biosynthesis of amino acids
Arrhythmogenic right ventricular cardiomyopathy (ARVC)
Aldosterone−regulated sodium reabsorption
Alcoholism
Adrenergic signaling in cardiomyocytes
ABC transporters
0.4
0.6
0.8
1.0
Rich factor
qvalue
1.00
0.75
0.50
0.25
0.00
Gene_number
20
40
60
E120 vs E65 DEG enriched KEGG pathway
