# Supplementary material for: Comprehensive Transcriptome Analysis of Hair Follicle Morphogenesis Reveals That lncRNA-H19 Promotes Dermal Papilla Cell Proliferation through the Chi-miR-214-3p/β-Catenin Axis in Cashmere Goats
Source: Int J Mol Sci. 2022 Sep 2;23(17):10006. doi: 10.3390/ijms231710006 (PMC9456307; doi:10.3390/ijms231710006)
Supplement: Supplementary file 1 [file ijms-23-10006-s001.zip › ijms-1812373-supplementary/Supplementary Figure legends.pdf]

Supplementary Figure legends

Fig.S1 Up- or down-DE genes and ncRNAs were visualized using volcano plots (A)The Volcano Plot of the differentially expressed genes. (B) The Volcano Plot of the differentially expressed lncRNAs. (C) The Volcano Plot of the differentially expressed miRNAs. up- or down-DE ncRNAs and genes were visualized using volcano plots.

Fig.S2 Verification of the differentially expressed genes and ncRNAs of RNA-seq. (A) RT-qPCR of five randomly selected genes. (B) RT-qPCR of five randomly selected lncRNAs. (C) RT-qPCR of five randomly selected miRNAs. Values with letters a, b, and c are significantly different across columns ($P < 0.05$).

Fig.S3 KEGG analysis of the DEGs. (A) E90 VS E65 enriched KEGG pathways. (B) E120 VS E90 enriched KEGG pathways. (C) E120 VS E65 enriched KEGG pathways.

Fig.S4 The regulatory network of ceRNA. (A) E90 VS E65 ceRNA network. (B) E120 VS E90 ceRNA network. (C) E120 VS E65 enriched KEGG ceRNA network.

Fig.S5 The expression of *CTNNB1* gene was detected by RT-qPCR normalized to β-actin.
