# Supplementary material for: Comprehensive Transcriptome Analysis of Hair Follicle Morphogenesis Reveals That lncRNA-H19 Promotes Dermal Papilla Cell Proliferation through the Chi-miR-214-3p/β-Catenin Axis in Cashmere Goats
Source: Int J Mol Sci. 2022 Sep 2;23(17):10006. doi: 10.3390/ijms231710006 (PMC9456307; doi:10.3390/ijms231710006)
Supplement: Supplementary file 1 [file ijms-23-10006-s001.zip › ijms-1812373-supplementary/Table S1 and S2.pdf]

Table S1 Summary of data output for each sample (LncRNA libraries)

Sample name	Raw reads	Clean reads	Raw bases(G)	Clean bases(G)	Error rate (%)	Q20 (%)	Q30 (%)	GC content (%)
E120_1	95,136,750	94,162,346	14.27	14.12	0.01	97.99	94.87	48.39
E120_2	104,858,132	103,693,97	15.73	15.55	0.01	97.82	94.51	51.99
E120_3	109,873,850	108,854,76	16.48	16.33	0.01	97.91	94.75	51.99
E65_1	92,319,368	91,436,574	13.85	13.72	0.01	98.07	95.06	51.95
E65_2	95,265,442	94,404,738	14.29	14.16	0.01	98.1	95.11	52.43
E65_3	114,213,636	113,055,67	17.13	16.96	0.01	98.16	95.25	45.19
E90_1	91,771,374	90,803,258	13.77	13.62	0.03	97.33	92.59	48.6
E90_2	85,447,776	83,722,880	12.82	12.56	0.03	97.76	93.57	48.59
E90_3	85,950,416	84,254,962	12.89	12.64	0.03	97.69	93.43	50.76

Table S2 Summary of data output for each sample (miRNA libraries)

Sample	Raw Reads	Clean Reads	Bases	Error rate	Q20	Q30	GC content
E120_3	24,594,694	24,108,334	1.230G	0.01%	98.94%	96.06%	49.94%
E120_2	23,480,919	22,890,002	1.174G	0.01%	98.84%	95.82%	50.29%
E120_1	19,106,255	17,689,794	0.955G	0.01%	98.14%	94.35%	50.08%
E90_2	18,281,030	17,152,251	0.914G	0.01%	98.21%	94.56%	48.92%
E90_3	16,751,062	15,648,767	0.838G	0.01%	98.22%	94.64%	49.43%
E90_1	11,090,195	10,186,863	0.555G	0.01%	98.52%	95.33%	49.55%
E65_2	21,689,760	21,261,809	1.084G	0.01%	98.85%	95.82%	49.50%
E65_3	28,099,273	27,418,451	1.405G	0.01%	98.71%	95.35%	50.09%
E65_1	21,919,844	21,424,462	1.096G	0.01%	98.87%	95.84%	50.39%
