# Supplementary figures and images for: Comprehensive Transcriptome Analysis of Hair Follicle Morphogenesis Reveals That lncRNA-H19 Promotes Dermal Papilla Cell Proliferation through the Chi-miR-214-3p/β-Catenin Axis in Cashmere Goats
Source: Int J Mol Sci. 2022 Sep 2;23(17):10006. doi: 10.3390/ijms231710006 (PMC9456307; doi:10.3390/ijms231710006)

A

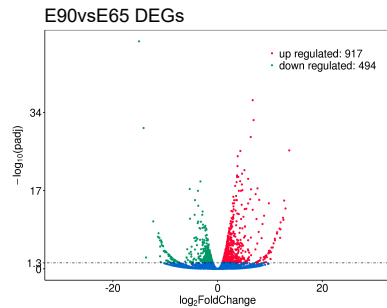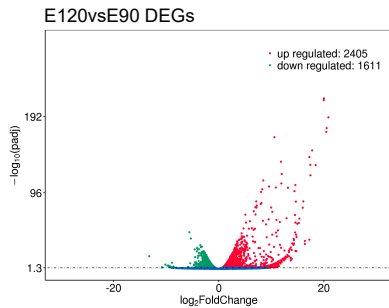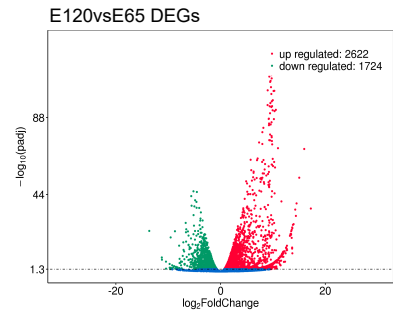

B

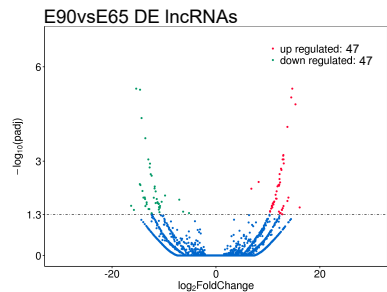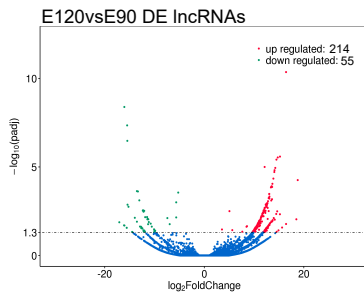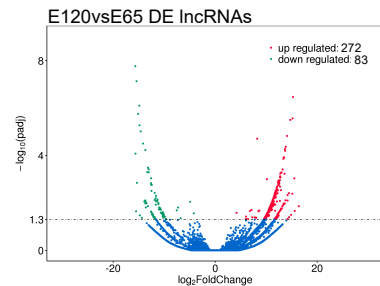

C

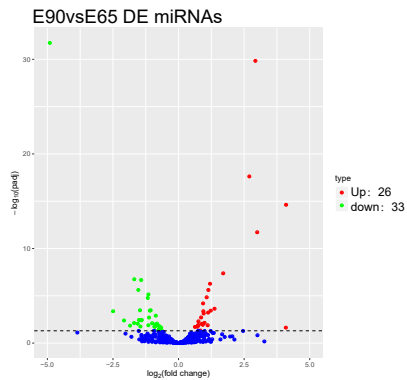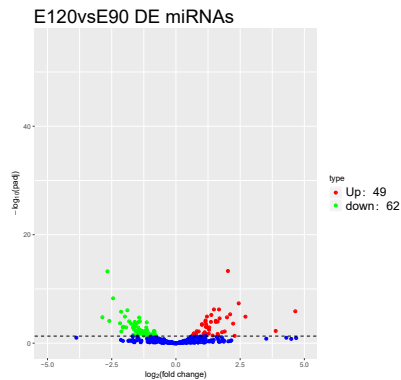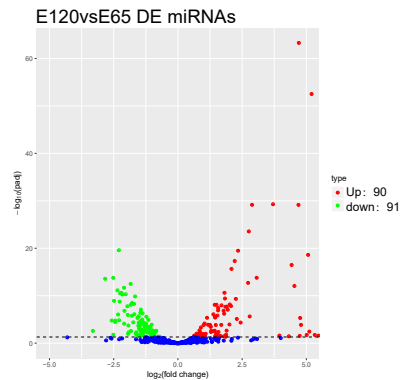

Supplement: Supplementary file 1 [file ijms-23-10006-s001.zip › ijms-1812373-supplementary/Fig.S1.pdf]

■ E65 ■ E90 ■ E120

A

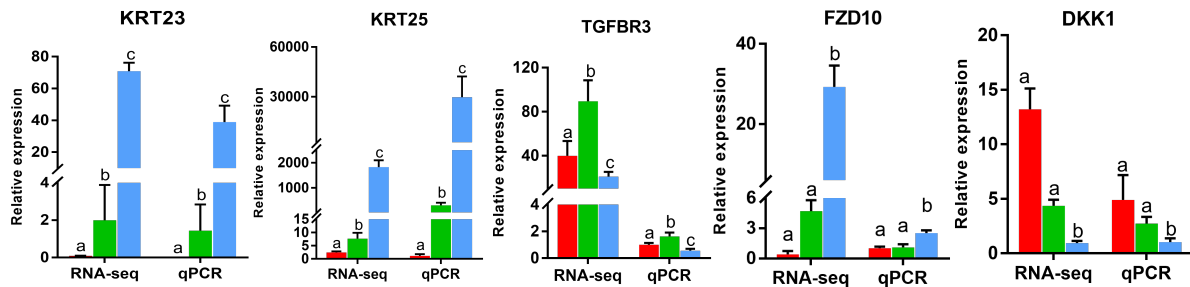

B

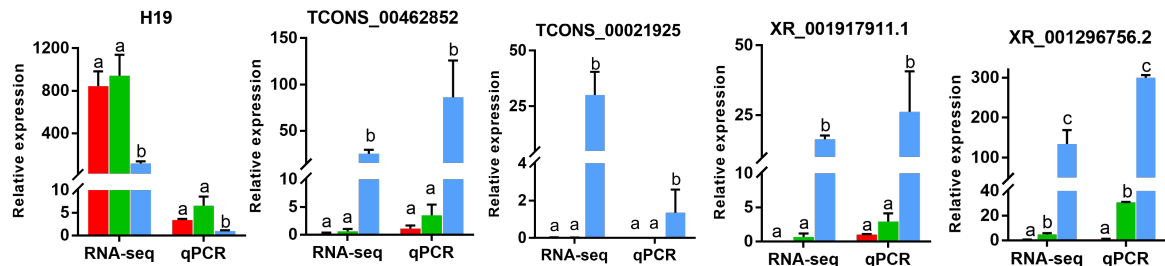

C

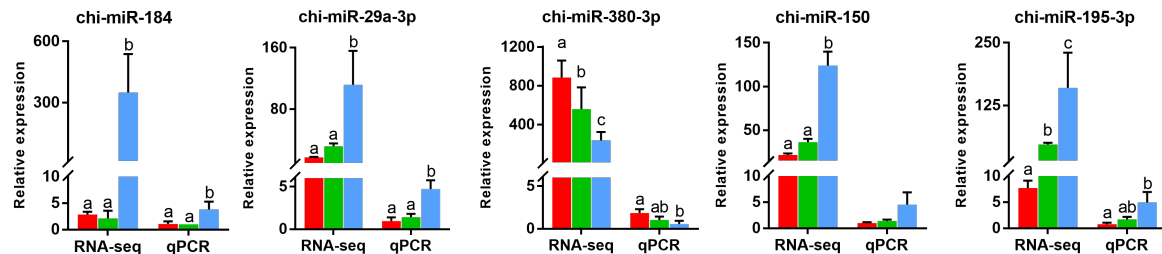

Supplement: Supplementary file 1 [file ijms-23-10006-s001.zip › ijms-1812373-supplementary/Fig.S2.pdf]

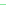 lncRNA  
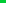 miRNA  
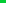 gene

[illegible]

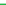 lncRNA  
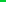 miRNA  
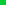 gene

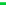 lncRNA  
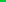 miRNA  
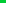 gene

Supplement: Supplementary file 1 [file ijms-23-10006-s001.zip › ijms-1812373-supplementary/Fig.S4.pdf]

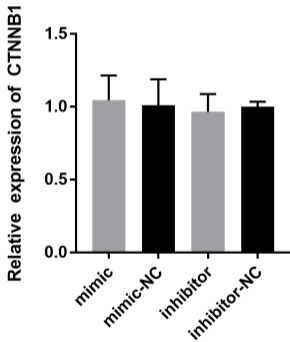

Supplement: Supplementary file 1 [file ijms-23-10006-s001.zip › ijms-1812373-supplementary/Fig.S5.pdf]
